# Graves’ disease: review of disease characteristics, burden, and treatments

**DOI:** 10.3389/fendo.2026.1884624

**Published:** 2026-08-18

**Authors:** Lesley-Ann Miller-Wilson, Erin Mandal, Kylle M. Tollefsen, Sarah Ronnebaum, Sofie Norregaard, Julie Chen

**Affiliations:** 1 Immunovant, Inc., Durham, NC, United States; 2 Thermo Fisher Scientific, Waltham, MA, United States; 3 Thermo Fisher Scientific, London, United Kingdom; 4 Department of Medicine, Division of Endocrinology, Gerontology, and Metabolism, Stanford University Medical Center, Palo Alto, CA, United States

**Keywords:** burden of disease, costs, epidemiology, Graves’ disease, quality of life, treatment patterns, unmet needs, Basedow's disease

## Abstract

Graves’ disease, the most common cause of hyperthyroidism, is a chronic autoimmune disorder that can lead to serious health problems. Treatment options for Graves’ hyperthyroidism have not changed in several decades and include antithyroid drugs (ATDs), radioactive iodine (RAI), and thyroidectomy. Few literature reviews comprehensively evaluating the burden of Graves’ disease, including epidemiological, clinical, humanistic, and economic outcomes, have been published since the American Thyroid Association and European Thyroid Association treatment guidelines for Graves’ disease were developed in 2016 and 2018, respectively. In this review, we evaluate the disease burden, treatment landscape, unmet needs, and research gaps among adult patients with Graves’ disease. Significant clinical and humanistic burden is observed in patients with Graves’ disease, including treated patients, compared to control populations. Across global survey studies, over 90% of clinicians preferred ATDs compared to RAI and thyroidectomy as the first-line treatment of Graves’ disease; however, a reported 37% to 76% of patients who achieve euthyroidism with a first-line course of ATDs will subsequently relapse. Although long-term use (>2 years) of low-dose ATDs can improve relapse rates compared with conventional ATD treatment (12–18 months), a subset of patients will not achieve sufficient control of their hyperthyroidism with an initial course of ATDs and approximately one-fifth of patients will experience adverse effects with long-term ATDs. Limited evidence on the economic burden of Graves’ disease was identified. Results suggest ATDs or thyroidectomy are both more cost-effective than RAI. In conclusion, patients with Graves’ disease experience substantial clinical and humanistic disease burden that current treatment options do not adequately address. While this targeted literature review was guided by a protocol, qualitative evidence synthesis is limited due to its nonsystematic nature. Further evaluation based on robust, real-world evidence studies is needed to understand the long-term impact of Graves’ disease and care needs throughout a patient’s lifetime.

## 1 Introduction

Graves’ disease (GD) is an autoimmune disorder affecting multiple organs, including the thyroid, heart, eyes, liver, bone, skeletal muscle, and skin, and is the most common cause of hyperthyroidism (1, 2). The overall prevalence of GD is estimated to be 1% to 1.5%, with women being approximately 4–10 times more likely to develop GD than men (1, 3, 4). Common signs and symptoms of GD include tiredness, weight loss, anxiety, trembling, tachycardia, heat intolerance, enlargement of the thyroid gland (goiter), alterations in menstrual cycles, erectile dysfunction or decreased libido, and frequent bowel movements; patients with GD may also experience extrathyroidal manifestations, such as thyroid eye disease (TED), skin manifestations, cardiovascular complications, and osteoporosis (1, 2, 5). The symptoms and manifestations experienced by patients with GD result in a reduced health-related quality of life (HRQoL), even after receiving treatment (6). Treatment options for GD have remained the same for several decades and include antithyroid drugs (ATDs; most commonly methimazole [MMI]), approved by the United States (US) Food and Drug Administration (FDA) in 1950; radioactive iodine (RAI), approved by the US FDA in 1971; and thyroidectomy, which has been used for more than 80 years to treat hyperthyroidism, but is not US FDA-approved for the treatment of GD (1, 2, 7–10). Although ATDs have increasingly become the first-line treatment of choice (7), relapses following first-line ATD treatment are common (11). Thyroidectomy and RAI will almost always result in permanent hypothyroidism requiring long-term thyroid hormone replacement therapy and may also lead to other complications, such as hypoparathyroidism and/or recurrent laryngeal nerve damage due to thyroidectomy and worsening or new onset TED associated with RAI (1, 12–15).

Published evidence describing the comprehensive burden of GD, including epidemiologic, clinical, humanistic, and economic considerations is lacking. The objective of this review is to provide a complete view of the burden of GD based on recent data, including unmet patient needs, as well as a review of the current treatment landscape and research gaps.

## 2 Methodology

### 2.1 Data sources

Databases of published literature (Embase, MEDLINE, Cochrane Library, and Econlit) were searched utilizing a combination of free-text searches and medical subject headings (MeSH) terms via the OvidSP^®^ platform on August 2, 2024 (**Supplementary Table 3**–**9**). Manuscripts published from 2019 onwards and conference abstracts published from 2021 onwards were eligible for inclusion. Grey literature sources were also evaluated, including conference abstracts (American Thyroid Association [ATA], British Thyroid Foundation, European Thyroid Association [ETA], International Society for Pharmacoeconomics and Outcomes Research), reference lists of relevant studies, and Clinicaltrials.gov. During manuscript preparation, peer-reviewed full-text articles describing the same study as a previously included conference abstract were replaced in the evidence synthesis.

### 2.2 Study selection

This targeted literature review was guided by a protocol, including pre-defined population, interventions and comparisons, outcomes, and study design (PICOS) selection criteria (**Supplementary Table 2**). Publications in English that evaluated adults (≥18 years) with GD and contained information on the following topics were selected for inclusion: epidemiological, clinical, humanistic, and economic outcomes; real-world treatment patterns; and ongoing clinical trials. Real-world prospective or retrospective studies, clinical trials, economic models, and guidelines were included. Since there has been little advancement in the treatment of Graves’ hyperthyroidism in recent years, systematic literature reviews and meta-analyses were used as a source of pooled quantitative data for primary studies published prior to 2019. The authors focused primarily on evidence from the US, Canada, and Europe given the global influence of Western countries on drug development and regulatory approvals; however, global evidence was also considered.

### 2.3 Review procedure

An independent researcher completed title/abstract and full text screening, identified studies for inclusion based on the PICOS selection criteria, and extracted data into an Excel-based data-extraction template. A second independent researcher performed standard quality checks on approximately 10% of randomly selected references during the abstract and full text screening to verify results. A formal risk of bias and quality assessment of included references was not conducted.

A total of 2,152 studies were screened at the title/abstract level; of these 83 studies were included and an additional 24 clinical trial records were identified and included from ClinicalTrials.gov (**Supplementary Figure 1**).

## 3 Epidemiological and clinical burden

### 3.1 Prevalence/incidence

Evidence describing the contemporary epidemiology of GD was limited. No citations published since 2019 were identified that estimated the prevalence of GD, and inconsistent incidence trends were reported across studies. Treatment guidelines published by the ATA in 2016 (2) and the ETA in 2018 (1) reported that the prevalence of hyperthyroidism was approximately 1.2% to 1.6%, with GD being the most frequent cause. More recent evidence was available regarding the incidence of GD, with estimates ranging from 8.3 per 100, 000 people in Denmark in 2018 to 66 per 100, 000 people in the United Kingdom (UK) in 2019 (16–18). Directional trends were inconsistent across studies, with decreasing incidence rates in Denmark (from 1995 to 2018) and Italy (from 2013 to 2022) and increasing rates in the UK (from 2000 to 2019) (16–18). In Italy, study authors postulated that a regional iodine prophylaxis campaign may have contributed to the decreasing incidence of GD, although the association of iodine prophylaxis and GD incidence has not been consistently observed across other studies (16). In the UK, the increase of incidence was largely driven by a rise in diagnoses in individuals ≥80 years of age (17). The Danish study did not discuss factors impacting incidence trends (18).

### 3.2 Mortality

Limited evidence on the mortality of GD was identified, and no comparisons were performed across geographic regions (19, 20). In a retrospective cohort study conducted in the UK, patients with GD had a 23% increased risk of all-cause mortality compared with matched controls (19). Furthermore, patients with GD and persistently low serum thyroid-stimulating hormone (TSH) one year after diagnosis had a 55% increased risk of all-cause mortality compared to patients with GD and normal serum TSH (19). Results from a meta-analysis demonstrated that, among patients with differentiated thyroid carcinoma, the overall mortality rate was nearly 3 times higher among patients with GD compared to those without GD (20).

### 3.3 Risk factors

Demographic, behavioral, and genetic variables are all implicated as risk factors for GD; however, the relative importance of these risk factors is unclear based on recent literature (1, 17, 21–24). Identified risk factors for the development of GD included female gender, smoking, presence of other autoimmune disorders, and certain gene polymorphisms (including polymorphisms in tumor necrosis factor-α, interleukin [IL]-1 [IL-1], IL-6, TSH receptor, and human leukocyte antigen class II genes) (1, 17, 21–23). Results from a meta-analysis of 110 studies (22, 403 patients with GD) found that 81% (95% CI: 79-83) of patients were female and the mean age of patients at diagnosis was 39.8 years (95% CI: 38.4-41.1) (25). Results from another meta-analysis (9 studies; 2, 258 participants, including 1, 051 patients with GD and 1, 207 healthy controls) suggested that stressful life events before GD diagnosis may be an environmental trigger, especially among younger women (24).

### 3.4 Subclinical hyperthyroidism

Subclinical hyperthyroidism is characterized by suppressed TSH, while serum free thyroxine (FT4) and total triiodothyronine (T3) or free T3 (FT3) levels are within reference ranges (1, 2). This condition is associated with poor outcomes, including increased risk of adverse cardiovascular events and mortality (1, 2). Patients with GD and subclinical hyperthyroidism may progress to overt hyperthyroidism or spontaneously remit (1, 2). The ATA and ETA guidelines recommend ATD treatment of subclinical hyperthyroidism among patients with GD aged ≥65 years whose serum TSH levels are persistently <0.1 mIU/L; ATDs may also be considered in patients <65 years of age with subclinical hyperthyroidism and certain risk factors, such as cardiac disease or osteoporosis (1, 2).

Limited data suggest some patients may experience transient subclinical hyperthyroidism after ATD or RAI treatment. In a clinical trial comparing short-term (12–18 months) vs long-term (60–120 months) MMI treatment, transient subclinical hyperthyroidism was observed in 10.8% (13/120) of patients after MMI was discontinued in the short-term group vs 9.3% (11/118) of patients in the long-term group (26). In a real-world setting evaluating patients for up to one year after RAI therapy, transient subclinical hyperthyroidism was observed in up to 22.8% (165/723) of patients (27). In patients who received ATDs alone as a post-RAI treatment strategy, up to 22.7% (42/185) of patients experienced transient subclinical hyperthyroidism (27). No additional studies describing the impact of GD treatments on the prevalence of sublinical hyperthyroidism were identified.

### 3.5 Extrathyroidal manifestations and comorbidities

Thyroid eye disease, also referred to as Graves’ orbitopathy (GO), is a common extrathyroidal manifestation of GD that affects approximately 30% to 40% of patients with GD (21, 25, 28). Common clinical manifestations related to TED, as reported by a meta-analysis (57 studies; 26, 804 patients with GD), included lid retraction (57% [95% CI: 0.39-0.74]), proptosis (57% [95% CI: 0.48-0.65]), diplopia (36% [95% CI: 0.24-0.48]), and ocular hypertension (13% [95% CI: 0.06-0.19]) (28). In addition, patients with GD and TED were significantly more likely to have at least one additional autoimmune disorder than those without TED (18.9% vs 15.6% of patients; p < 0.0201), based on an Italian, prospective analysis of 3,209 consecutive patients with GD from 1993 to 2010 (21).

Patients with GD had a significantly increased risk of several comorbidities compared to matched controls, including cardiovascular conditions, diabetes, dementia, and thyroid cancer, as well as increased use of osteoporosis treatments (**Table 1**) (19, 29–33).

**Table 1:** Summary of real-world evidence studies comparing the rates of comorbidities among patients with GD vs controls.

Author year	Study design (years); country	Patient population	Key results
Studies comparing patients with GD vs matched controls			
Okosieme 2019 (19)	Retrospective cohort study (1998–2013); UK	• GD (n=4,189) • Matched controls (n=16,756)	• A significantly higher risk of the following comorbidities was observed among patients with GD compared with controls; all data reported as aHR (95% CI) -MACE: 2.47 (2.16–2.81); *p* < 0.0001 -Atrial fibrillation: 2.67 (2.25–3.15); *p* < 0.0001 -Acute coronary syndrome: 1.65 (1.09–2.51); *p* = 0.018 -Heart failure: 1.78 (1.42–2.23); *p* = 0.001 -Ischemic stroke: 1.60 (1.11–2.30); *p* = 0.012 -Diabetes: 1.46 (1.24–1.71); *p* = 0.001 -Thyroid cancer: 6.67 (2.18–20.41); *p* = 0.001
Song 2021 (30)	Retrospective cohort study (2005–2012); Republic of Korea	• GD treated with ATDs for ≥24 months (n=4,593) • Matched controls (n=45,930)	• A significantly higher risk of diabetes was observed among patients with GD compared with controls; data reported as aHR (95% CI): -1.18 (1.1–1.28); *p* < 0.001
Song 2021 (29)	Retrospective cohort study (2005–2012); Republic of Korea	• GD treated with ATDs for ≥24 months (n=16,882) • Matched controls (n=84,263)	• A significantly higher risk of heart failure was observed among patients with GD compared with controls; all data reported as aHR (95% CI) -ATD-treated patients vs controls: 1.28 (1.2–1.36); *p* < 0.001 -Subset of patients who received subsequent RAI therapy after ATDs (n=366) vs controls: 1.49 (1.01–2.19); *p* = 0.044
Folkestad 2020 (31)	Registry study (1995–2013); Denmark	• GD (n=35,522) • Matched controls (n=142,088)	• A significantly higher risk of dementia was observed among patients with GD compared with controls; data reported as aHR (95% CI) -1.13 (1.06–1.21); *p* value not reported. Study authors noted that an aHR>1 is accepted as significant • A significantly higher risk of dementia compared with matched controls was also observed among a broader population with hyperthyroidism due to GD or other causes
Khamisi 2023 (33)	Retrospective cohort study (2005–2019); Sweden	• GD (n=2,134) • Matched controls (n=21,261)	• After a median follow-up of 15.2 years, patients with GD compared with controls had the following; all data reported as aHR (CI): -Significantly higher rates of osteoporosis treatments: 2.74 (1.85-4.03); *p* value not reported -No difference in osteoporosis-related fractures: 0.99 (0.86–1.15); *p* value not reported Note: confidence level was not explicitly stated by study authors
Study comparing patients with subclinical hyperthyroidism vs euthyroidism			
Liu 2024 (32)	Meta-analysis (9 cohort studies)	• Community-derived participants (n=49,218)	• A significantly higher incidence of dementia was observed among participants with subclinical hyperthyroidism compared to patients with euthyroidism: RR = 1.38 (95% CI: 1.09–1.74); *p* = 0.006

aHR, adjusted hazard ratio; ATD, antithyroid drug; CI, confidence interval; GD, Graves’ disease; MACE, major adverse cardiovascular event; RAI, radioactive iodine; RR, risk ratio.

## 4 Humanistic burden

Validated patient-reported outcome (PRO) tools used to evaluate the HRQoL and symptom burden in patients with GD or TED included the 36-item Short-Form Health Status (SF-36) (34, 35), MD Anderson Symptom Inventory (36), Mental Fatigue Scale (37, 38), and Comprehensive Psychological Rating Scale (37, 38). Assessments specific to thyroid disease were also used, including the Thyroid-Related Patient-Reported Outcome (ThyPRO) (6, 34, 35, 39) and Graves Ophthalmopathy Quality of Life (GO-QOL) (40–43) instruments.

Impaired HRQoL was consistently observed among patients with GD, including patients treated with ATDs, RAI, or thyroidectomy, compared to controls (**Table 2**) (6, 34, 35, 37, 38). When considering different treatment modalities, patients treated with ATDs experienced improvements in HRQoL compared to pre-treatment periods, and these patients generally had better HRQoL compared to patients treated with RAI or thyroidectomy (6, 38, 39). There is limited evidence regarding thyroid-specific symptoms and patient-reported improvements in HRQoL associated with RAI and total thyroidectomy (12, 36).

**Table 2:** Summary of real-world evidence studies reporting on patient-reported outcomes among patients with Graves’ hyperthyroidism.

Author year	Study Design (years); country	Patient population	Key results
Meling Stokland 2024 (6)	Retrospective follow-up performed 25 years after completion of a randomized trial evaluating ATDs (1997 to 2001); Norway	• GD (N = 182), including the following subgroups: -ATD-treated (n=49) -RAI-treated (n=51) -Thyroidectomy-treated, subtotal or total (n=21) -Patients with spontaneous hypothyroidism (n=15)	• Patients treated with ATDs had significantly (*p* < 0.05) worse physical symptoms as measured by the ThyPRO (including goiter, hyperthyroid, hypothyroid, and eye-related symptoms) compared to Danish general population norms • Patients with GD and hypothyroidism (due to treatment with RAI or thyroidectomy, or spontaneous hypothyroidism) had significantly (*p* < 0.05) worse scores on most ThyPRO subscales compared to Danish general population norms and to ATD-treated patients • Patients treated with ATDs had similar or significantly (*p* < 0.05) better scores on ThyPRO subscales compared to patients treated with RAI or thyroidectomy
Calissendorff 2023 (34) Torring 2019 (35)	Pragmatic trial (2003–2013); Sweden	• GD (n=1,186)	• Men and women with GD experienced significantly (*p* < 0.01) worse scores on several SF-36 domains compared with Swedish population norms, including mental health, vitality, and (for women only) bodily pain • Men and women with GD experienced significantly (*p* < 0.05) worse scores on most ThyPRO domains compared with Danish population norms (all domains except tiredness) • Patients treated for GD with ATD, RAI or thyroidectomy had worse scores on most ThyPRO domains compared to Danish population norms -ATD-treated: all domains were significantly worse (*p* < 0.05) in treated patients except tiredness and emotional susceptibility -RAI-treated: all domains were significantly worse (*p* < 0.05) in treated patients -Thyroidectomy: all domains were significantly worse (*p* < 0.05) in treated patients except tiredness and emotional susceptibility • Patients treated with RAI generally had significantly worse (*p* < 0.05) ThyPRO domain scores compared to patients treated with ATDs or thyroidectomy
Holmberg 2024 (37) Johansson 2023 (38)	Prospective, longitudinal, case-controlled study (CogThy study; 2011-2019); Sweden	• Newly diagnosed GD (n=65) • Matched controls (n=65)	• Patients with GD during a hyperthyroid phase had significantly worse mean (SD) scores for mental fatigue, depression, and anxiety compared to controls: -MFS: 17.5 (6.3) vs 5.6 (5.5); *p* < 0.001 -CPRS depression scale: 7.5 (4.1) vs 1.9 (1.9); *p* < 0.001 -CPRS anxiety scale: 8.3 (4.2) vs 3.0 (2.5); *p* < 0.001 • Patients with GD treated with ATDs for 15 months had significantly worse median (IQR) scores for depression and anxiety compared to controls: -CPRS depression scale: 2.5 (1.5-5.0) vs 1.5 (0.5-3.5); *p* < 0.05 -CPRS anxiety scale: 4.0 (2.5-7.5) vs 3.0 (1.5-5.0); *p* < 0.05 • Patients with GD treated with ATDs for 15 months had significantly improved mean (SD) scores for depression and anxiety compared to patients with GD during a pre-treatment, hyperthyroid phase: -MFS: 9.5 (6.4) vs 17.5 (6.3); *p* < 0.001 -CPRS depression scale: 3.9 (4.2) vs 7.5 (4.1); *p* < 0.001 -CPRS anxiety scale: 5.0 (3.9) vs 8.3 (4.2); *p* < 0.001
Gunn 2022 (36)	Prospective cohort study (2015–2020); US	• GD; patients underwent total thyroidectomy (n=85)	• Patient-reported burden in the MDASI Thyroid Symptom Score and the QoL Symptom Score improved following thyroidectomy, both in the short- and long-term: -Thyroid Symptom Score, short-term: RR = 0.55 (CI: 0.42–0.72) -Thyroid Symptom Score, long-term: RR = 0.59 (CI: 0.44–0.79) -QoL Symptom Score, short-term: RR = 0.57 (CI: 0.40–0.81) -QoL Symptom Score, long-term: RR = 0.43 (CI: 0.28–0.65) Note: confidence level was not explicitly stated by study authors and *p* values were not reported
Aung 2019 (12)	Retrospective, cohort study (2006–2015); UK	• GD; patients treated with RAI (n=576)	• No differences in frequency of patient-reported thyroid symptoms were observed before or after RAI therapy, or based on treatment success or failure

ATD, anti-thyroid drug; CI, confidence interval; CogThy, Cognition in Thyroid Disease study; CPRS, Comprehensive Psychological Rating Scale; GD, Graves’ disease; IQR, interquartile range; MDASI, MD Anderson Symptom Inventory; MFS, Mental Fatigue Scale; QoL, quality of life; RAI, radioactive iodine; RR, rate ratio; SD, standard deviation; SF-36, 36-item Short-Form Health Status; ThyPRO, Thyroid-Related Patient-Reported Outcome; UK, United Kingdom; US, United States.

Among patients with TED, real-world and clinical trial-based evidence demonstrated that patients who received treatment for TED (including those treated with teprotumumab, methylprednisolone, sirolimus, and ophthalmic surgery) generally experienced improvements in HRQoL, as measured by the GO-QOL (41–45).

## 5 Economic burden

Limited economic evidence was identified in patients with GD; in total, eight studies representing patients from the US, the UK, Denmark, Sweden, and China were identified (12, 13, 46–51). Two studies were economic models, including a Swedish cost-utility analysis evaluating ATD, RAI, and thyroidectomy (total or partial thyroidectomy not specified) over an 8-year time horizon and a US-based cost-effectiveness model evaluating the lifetime costs of RAI versus total thyroidectomy (49, 51). For initial treatment of GD, the Swedish cost utility analysis demonstrated that ATDs dominated RAI (i.e., ATDs were less costly and more effective) (51). Thyroid surgery was more costly and more effective than ATDs and was more cost-effective than RAI (51). The US-based model similarly found that total thyroidectomy was highly cost-effective compared with RAI therapy for the treatment of a 40-year old woman with medication refractory GD (49).

Among six studies reporting healthcare resource use (HCRU) and costs, findings were inconsistent and highlight the lack of available literature in this space (12, 13, 46–48, 50). Among available GD treatment modalities, a Danish multicenter study showed that RAI had the lowest life time costs, followed by ATDs and surgery (surgery type not specified); cost of treatment monitoring was not included (46). In two Chinese studies, however, 10-year cumulative direct healthcare costs were numerically highest for RAI, followed by ATDs and thyroidectomy for patients with GD both in the first-line setting and for patients with persistent or relapsed GD (47, 48). No studies describing the contribution of systematic manifestations of GD to overall HCRU or costs of GD were identified. Further evaluation across geographic regions on the long-term costs of treatment, monitoring, and contribution of systematic manifestations of GD is needed.

Additional studies evaluated the short-term costs and HCRU of specific treatments and complications (12, 13, 50). Among patients treated with ATDs, a Chinese retrospective study found that patients with agranulocytosis, a rare and serious side effect, had significantly increased median hospital costs ($2,811 vs $759; p < 0.001) and a significantly increased median length of stay (12 days vs 6 days; p < 0.001) compared to patients without agranulocytosis (50). Among patients who underwent thyroidectomy, a US study demonstrated that HCRU (including length of stay, return visits to the operating room, emergency department visits, and readmissions) did not differ significantly between patients who were or were not in compliance with ATA guidelines (13).

## 6 Real-world treatment patterns

Real-world evidence demonstrated that ATD use has been increasing globally over the past several decades, whereas the use of RAI and thyroidectomy has been decreasing (7, 52–57). Results from a retrospective analysis of US claims data in adults with GD (N = 8, 217) found the proportion of patients receiving first-line ATD treatment rose from approximately 45% in 2005 to 65% in 2013 (52). Meanwhile, throughout the study period, the proportion of patients using first-line RAI decreased from approximately 45% to 25%, and the use of thyroidectomy remained stable (<10% of patients used first-line thyroidectomy) (52).

In accordance with changing treatment patterns, recent global surveys of endocrinologists and a discrete choice experiment evaluating preferences of patients and clinicians in Denmark indicate a clear consensus for ATDs as the preferred first-line treatment (7, 54–57). For uncomplicated GD, ATDs were the preferred first-line choice among 91.5% of endocrinologists who participated in a 2023 global survey (N = 1, 252), 95% of endocrinologists from a 2022–2023 UK-based survey (N=158), and 95% of endocrinologists from a 2022 Brazilian survey (N = 573) (7, 54, 57). In the global survey, the two most important reasons for preferring ATDs as primary therapy were a desire to avoid hypothyroidism and a desire to achieve remission (7). A Dutch discrete choice experiment was conducted between August 2019 and July 2020 to evaluate treatment preferences and trade-offs made by patients with GD (N = 286) and clinicians (N = 61) when choosing between different treatment options for GD, with results demonstrating that both patients and clinicians significantly (p < 0.05) preferred ATDs over surgery, and patients significantly (p < 0.05) preferred ATDs over RAI (55). Remission rate was the most important factor determining treatment choice, explaining 37% of choices in patients and 35% of choices in clinicians (55).

## 7 Treatment outcomes

### 7.1 Real-world evidence

Although several studies evaluating real-world treatment outcomes with ATD, RAI, and thyroidectomy were identified, definitions of outcomes (e.g., treatment success, failure, and remission) and duration of ATD use among study participants were heterogeneous, which limited meaningful qualitative comparisons between studies.

In general, patients were less likely to achieve remission on ATDs (approximately 30% to 60%) than on RAI therapy or thyroidectomy (both >80% to 90%) (18, 53, 58, 59). In a US claims-based study evaluating patients with GD who underwent first-line treatment between 2005 and 2013 (N = 4, 661), the rate of treatment success (defined as not requiring subsequent treatment after first-line treatment) was 50% for ATDs, compared with 93% for RAI and 99% for thyroidectomy (58). Similarly, results from a Swedish pragmatic trial evaluating patients with newly diagnosed GD from 2003 to 2005 (N = 1,186) found that remission rates (defined as euthyroidism 3 to 6 months after last intervention) were lower in patients who received first-line ATD treatment (45.3%) than in those who received first-line RAI (81.5%) or thyroidectomy (96.3%) (59).

**Figure 1:**
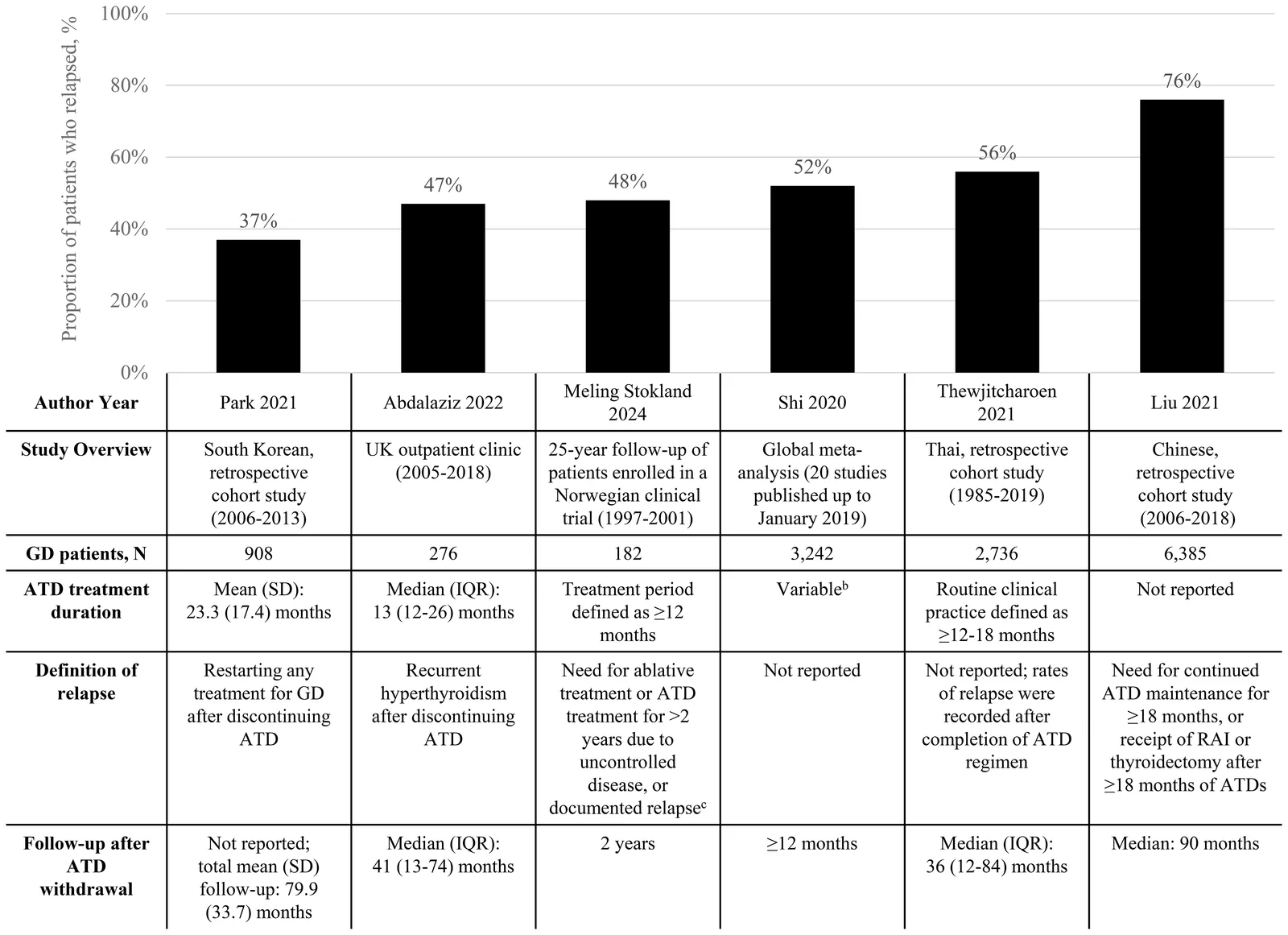
Proportion of patients with GD who relapsed after ATD withdrawal^a^. ATD, antithyroid drug; GD, Graves’ disease; IQR, interquartile range; RAI, radioactive iodine; UK, United Kingdom. ^a^ Study methodologies, definitions of relapse, ATD treatment durations, and follow-up periods varied across studies. Direct comparisons of relapse rates across studies should not be made. ^b^ Among 22 patient cohorts evaluated from 20 included studies in the meta-analysis, ATD duration was <12 months in 8 cohorts, between 12 and 24 months in 10 cohorts, and >24 months in 4 cohorts. ^c^ Definition provided by study authors was for persisting or relapsing disease activity.

A reported 37% to 76% of patients who successfully completed a course of first-line ATDs experienced a relapse of hyperthyroidism after stopping treatment (**Figure 1**) (6, 11, 48, 53, 60, 61). Time to relapse data were recorded in two publications, including a retrospective Korean cohort study, which reported a mean time of 16.5 months from ATD withdrawal to relapse, and a cohort study from Hong Kong, which reported a median time of 21 months (48, 61). The risk of relapse after stopping ATDs appears to be directly impacted by the duration of ATD treatment. Among patients with >1 year of follow-up after stopping ATD in the South Korean retrospective cohort (n=908), the rate of relapse decreased significantly with increasing ATD treatment duration (p = 0.003), with rates of 42.4%, 38.5%, 33.8%, 31.7%, 30.2%, 27.8%, and 19.0% for those on ATDs for <1 year, 1–2 years, 2–3 years, 3–4 years, 4–5 years, 5–6 years, and >6 years before withdrawal, respectively (61). Furthermore, evidence suggested that all patients who relapse after ATD withdrawal will do so within 10 years after stopping treatment (61).

### 7.2 Clinical trial-based evidence

Recent clinical trial–based evidence evaluating treatments for Graves’ hyperthyroidism generally focused on ATDs, either alone or in combination with an adjunctive or supplemental treatment. The identified trials evaluated long-term ATD use (26, 62–67), as well as ATDs in combination with vitamin D supplementation (39), selenium supplementation (68), or low-dose methotrexate (69).

Clinical trials assessing long-term ATD use in the US, Canada, and Europe were limited. However, in an Iranian clinical trial evaluating sustained euthyroidism in patients who discontinued ATD after a median of 18 months vs those who continued low-dose MMI for an additional 36–120 months (N = 258), relapse rates were lower among patients who continued MMI (**Table 3**) (26, 65). In another Iranian trial involving 59 patients who received MMI therapy for a mean of 14 years, those who subsequently discontinued MMI had an increased risk of recurrent hyperthyroidism and relapse six years later compared with patients who chose to continue treatment (62). Long-term ATD use was also reported to improve outcomes in patients following RAI. In a third Iranian trial enrolling patients who experienced a recurrence of hyperthyroidism after RAI treatment, those who subsequently received long-term MMI therapy (up to 5 years) had a higher rate of euthyroidism compared to patients who received a second dose of RAI (64).

**Table 3:** Summary of clinical trials evaluating long-term ATD use among patients with GD.

Author year	Trial and patient characteristics	Key study definitions	Key relapse or remission results
Lertwattanarak 2022 (66)	• Thai, 36-month, randomized, prospective clinical trial • Patients newly diagnosed with GD received MMI for ≥12 months followed by low-dose MMI for ≥6 months • Patients were randomized to discontinue low-dose MMI (n=92) or continue low-dose MMI (n=92)	• Remission defined as normal T3, FT4 and TSH levels that are stably controlled with low-dose MMI • Recurrent hyperthyroidism defined as elevated serum total T3 and/or FT4 levels and suppressed serum TSH levels	• Continuation of low-dose MMI therapy significantly decreased the risk of recurrent hyperthyroidism (HR = 0.26 [95% CI: 0.10–0.70]; *p* = 0.007) • Cumulative rates of recurrent hyperthyroidism were significantly lower in the continuation vs discontinuation group for all time points assessed (at 36 months [final time point]: 11.0% vs 41.2%; *p* < 0.01*)*
Azizi 2024 (26); Saadat 2024 (65)	• Iranian, 132-month, randomized clinical trial • Patients were treated with MMI for a median of 18 months and randomized to discontinue MMI (n=128; “conventional MMI group”) or continue MMI for an additional 36 to 120 months (n=130; “long-term MMI” group)	• Remission defined as ≥1 year of euthyroidism after discontinuing ATDs • Relapse defined as suppressed serum TSH with elevated FT4 or T3 after period of remission	• At 132 months follow-up, outcomes in the conventional vs long-term MMI group were:-Relapse rate: 54% vs 19%-Remission rate (i.e., euthyroidism): 44% vs 78% • At 84 months of follow-up, the hazard of recurrence in the conventional MMI group was 16.2 times greater than in the long-term MMI group (HR = 16.23 [95% CI: 4.67-56.48]; *p* < 0.001)
Azizi 2021 (62)	• Iranian, long-term extension study • Enrolled patients with GD (N = 59) were initially treated with long-term MMI (mean duration: 14.2 [SD: 2.9] years) • Patients subsequently chose to either discontinue long-term MMI treatment (n=32) or continue long-term MMI treatment (n=27)	• Relapse characterized by development of overt hyperthyroidism (i.e., suppressed serum TSH and elevated FT4 and/or T3 levels)	• No patients who continued long-term MMI relapsed, whereas 19% of patients who discontinued long-term MMI relapsed within 6 years of follow-up (log rank *p* = 0.019)
Saadat 2022 (64)	• Iranian, 60-month, randomized clinical trial • All patients had relapsed after prior RAI and were randomized to receive an additional RAI dose (n=32) or long-term MMI for up to 5 years (n=32)	• Euthyroid defined as normal serum FT4, T3 and TSH concentrations	• At 60 months follow-up, 97% of patients in the long-term MMI group versus 25% in the RAI group were euthyroid

ATD, antithyroid drug; CI, confidence interval; FT4, free thyroxine; GD, Graves’ disease; HR, hazard ratio; MMI, methimazole; RAI, radioactive iodine; SD, standard deviation; T3, triiodothyronine; TSH, thyroid-stimulating hormone.

Although evidence suggests that long-term use of ATDs can reduce the risk of relapse, patients may experience adverse effects (1, 2). These may include minor allergic side effects; increased risk of birth defects if ATDs are used during pregnancy; and rare, but serious adverse effects, such as hepatitis, a lupus-like syndrome, and agranulocytosis (1, 2). Results from a meta-analysis evaluating trials published between 1950 and 2016 evaluating long-term ATD use (defined as >24 months) found that the complication rate with long-term ATDs was 19.1% (95% CI: 9.6–30.9), of which 1.5% were major complications (67).

**Figure 2:**
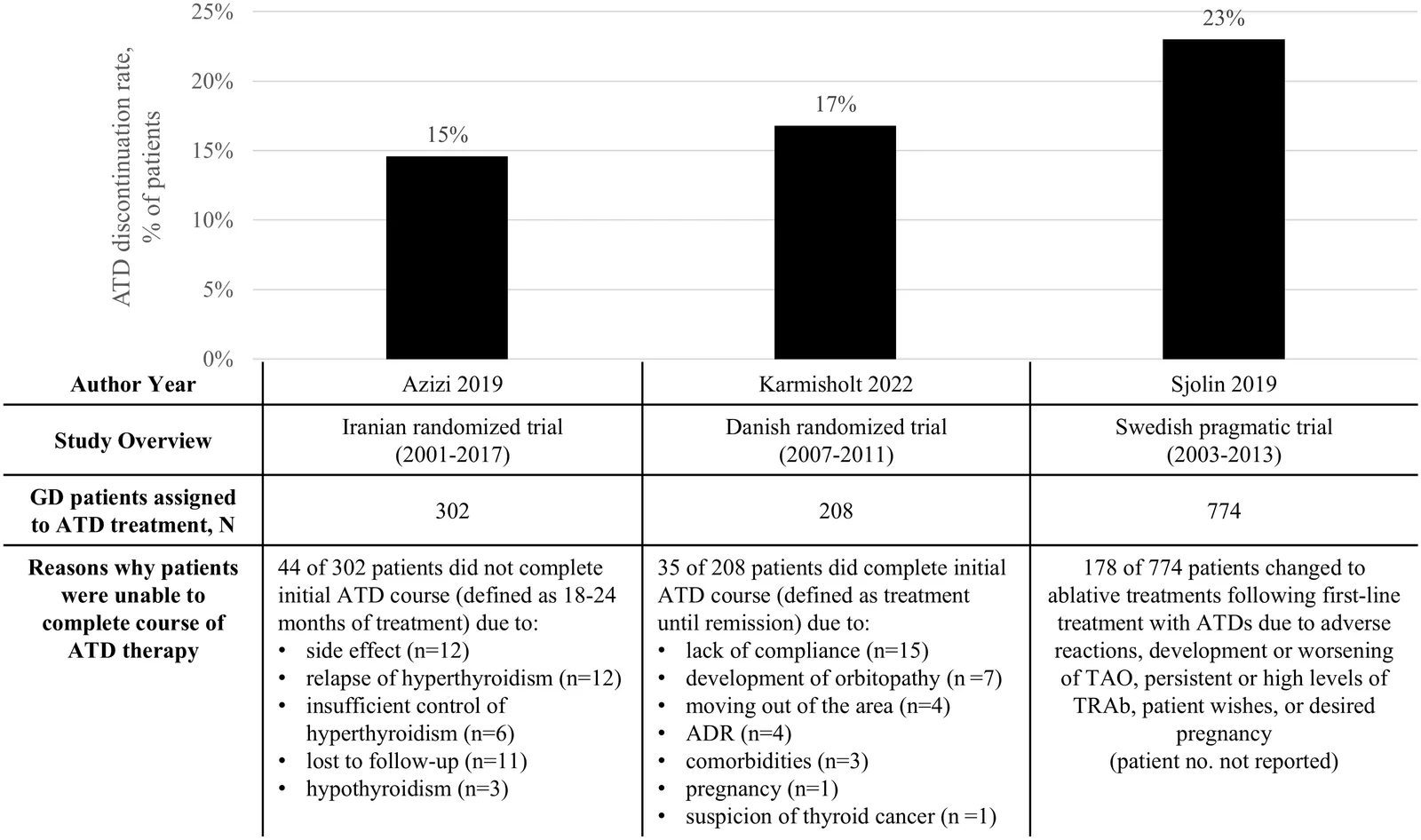
Patients with GD who discontinued an initial course of ATDs within clinical trials^a^. ADR, adverse drug reaction; ATD, antithyroid drug; GD, Graves’ disease; MMI, methimazole; TAO, thyroid-associated ophthalmology; TRAb, thyrotropin receptor antibody. ^a^ Study methodologies and reasons for discontinuation of initial ATD course varied across clinical trials. Direct comparisons of discontinuation rates across clinical trials should not be made.

Across three clinical trials reporting reasons for ATD discontinuation, 15% to 23% of patients with GD were unable to complete an initial treatment course of ATD treatment (**Figure 2**) (46, 59, 63). Patients discontinued treatment due to various reasons related to safety and effectiveness of ATDs, such as adverse effects or insufficient control of hyperthyroidism, or reasons related to clinical trial protocols, such as lack of compliance or lost to follow-up (46, 59, 63).

ATDs in combination with specific supplemental or adjunctive treatments may improve treatment outcomes, although further investigation is needed (68, 69). Selenium, which is recommended as monotherapy for mild, active TED in the 2022 ATA/ETA consensus statement on the management of TED and the 2021 European Group on GO (EUGOGO) treatment guidelines, has been evaluated in combination with ATDs for the treatment of GD (68, 70, 71). A meta-analysis evaluating randomized controlled trials published up to December 2022 (7 articles; 209 patients with GD) concluded that in patients treated with ATDs and selenium supplementation, thyroid function indicator levels (as measured by FT3, FT4, thyrotropin receptor antibodies, and thyroid peroxidase antibodies) were decreased compared to patients treated without selenium supplementation (68).

ATDs in combination with either adjunctive methotrexate or supplemental Vitamin D is not recommended in the ATA or ETA treatment guidelines (1, 2). Methotrexate in combination with MMI compared to MMI alone resulted in significantly higher rates of biochemical euthyroidism after 15 and 18 months of treatment, based on a Chinese, prospective, open-label randomized trial (69). Vitamin D supplementation with ATDs, however, was not found to significantly improve outcomes compared to ATDs in combination with placebo, based on Danish, double-blinded, randomized, multicenter trial (the DAGMAR Trial) (39).

## 8 Emerging treatments

ClinicalTrials.gov was searched for ongoing Phase 3 trials related to GD in June 2025.

### 8.1 Graves’ hyperthyroidism

We identified one academically sponsored Phase 2/3 trial evaluating treatments for Graves’ hyperthyroidism (NCT06068179) (72) and no industry-sponsored trials. The academic trial evaluated the efficacy and safety of mycophenolate mofetil (an immunosuppressor recommended for the treatment of TED/GO (71)) alone or combination with MMI for treatment of newly diagnosed GD (72). The primary outcome of this study was the remission rate at 12 months (defined as normal thyroid function and thyrotropin receptor antibody level) (72).

### 8.2 TED/GO

Twenty industry-sponsored Phase 3 trials were identified that are investigating targeted agents for the treatment of TED/GO (73–92) (**Supplementary Table 1**). The primary efficacy endpoints reported for these trials were all related to proptosis response. Evaluated treatments included those targeting FcRn, insulin-like growth factor 1 receptor (IGF-1R), and IL-6 (73–92). Specifically, batoclimab (73–75) and efgartigimod (91, 92) target FcRn; IBI311 (76, 77), MHB018A (78), linsitinib (79, 80), teprotumumab (83), VRDN-001 (also called veligrotug) (84–87), and VRDN-003 (88–90) target IGF-1R; and satralizumab (81, 82) targets IL-6.

## 9 Discussion

Patients with GD experience impaired HRQoL and substantial disease burden, including increased risks of mortality and several comorbidities (e.g., cardiovascular morbidities, diabetes, thyroid cancer, osteoporosis, and dementia) (2, 6, 19, 29–35, 37, 38). This disease burden is observed even among treated patients with GD, suggesting an unmet need for new treatment options that address the underlying causes of hyperthyroidism and potentially improve long-term outcomes related to mortality and extrathyroidal manifestations. Although we identified studies reporting on patients with transient subclinical hyperthyroidism following treatment for GD, additional research is needed on the prevalence and risks of subclinical hyperthyroidism in patients with persistent GD despite treatment.

New therapies have been developed for TED (70); however, no new treatments indicated specifically for Graves’ hyperthyroidism have become available in over 75 years. Among the available treatments for Graves’ hyperthyroidism (ATDs, RAI, and thyroidectomy), none target the underlying autoimmune response and pathogenic processes that cause hyperthyroidism (1, 2, 93). ATDs inhibit thyroid hormone production and are the most-used first-line treatment for GD (1, 52). However, ATDs have limited effectiveness: only approximately half of patients will experience remission (i.e., euthyroidism) with an initial course of ATDs and relapses are common following ATD withdrawal (6, 11, 48, 52, 53, 59–61). Nonetheless, ATDs remain the preferred first-line treatment for GD among both endocrinologists and patients worldwide, over RAI and thyroidectomy (7, 54–57). Although evidence from Asia and the Middle East indicates that long-term use (>2 years) of low-dose ATDs can reduce the risk of relapse, long-term use may not be appropriate for all patients due to adverse effects and risk of congenital birth defects if used during pregnancy (2, 61, 65, 67). Further evaluation on the risks and benefits of long-term ATD use across geographic regions is needed.

RAI therapy ablates thyroid tissue and thyroidectomy removes the target organ; however, patients will almost always require lifelong thyroid hormone replacement and neither treatment addresses the underlying autoimmune process of GD (1, 2, 93). In a global 2023 survey, these options were rarely selected as first-line therapy with some regional differences observed for RAI: 1.8% of European respondents selected RAI as first-line therapy compared to 13.1% of respondents in Africa and the Middle East and 11.1% in North America (7). Overall, use of RAI as first-line therapy has declined over the past several decades globally and in the US particularly, in part due to a desire to avoid permanent hypothyroidism (7, 52).

Although no ongoing Phase 3 clinical trials evaluating targeted treatments for Graves’ hyperthyroidism were identified in this review (search conducted up to June 2025), there are several novel therapies being investigated in predominately early stage trials and in in vitro and in vivo research studies (93). These emerging treatments have the potential to revolutionize GD management by targeting the causative molecular mechanisms of the disease, including immune or TSH receptor pathways (93). Drugs currently under investigation include monoclonal antibodies targeting CD20, CD40-CD40L signaling, BAFF, and FcRn; drugs targeting the TSH receptor signaling pathway; and drugs targeting the HLA-DR3 pathway (93). As of July 2026, we identified one agent that has received Investigational New Drug clearance from the US FDA for the treatment of Graves’ hyperthyroidism: imeroprubart, a fully human monoclonal antibody designed to target FcRn, received this designation in September 2024 to treat patients with GD who remain hyperthyroid despite using ATDs (94). Imeroprubart for the treatment of adult patients with GD is currently being evaluated in two phase 2b, randomized, double-blind placebo-controlled trials (NCT07018323 [estimated completion May 2027] (95) and NCT06727604 [estimated completion June 2028]) (96). The results of these ongoing phase 2b trials will determine the clinical impact of FcRn inhibition in patients with GD who remain hyperthyroid despite ATD use, such as meaningful improvements in disease control or remission rates. If these trials are successful, the addition of a new targeted treatment has the potential to expand the treatment paradigm for Graves’ hyperthyroidism.

### 9.1 Limitations

This targeted literature review was guided by a protocol, including pre-defined PICOS selection criteria and reproducible search strategies; however, limitations exist due to the nonsystematic nature of this review. First, a single reviewer performed screening, which may have resulted in erroneous exclusions of records eligible for inclusion in the review. To mitigate risks associated with a single-reviewer screening strategy, a random sample of 10% of the excluded abstracts and of the excluded full text articles were validated by a second, independent reviewer. Second, a formal assessment of risk of bias and study quality was not conducted. Included studies may vary in methodological rigor and have the potential for bias, thus qualitative evidence synthesis should be interpreted with caution.

Heterogeneity in study methods, outcome definitions (e.g., relapse, remission), duration of ATD use, and study follow-up periods also limited direct comparisons of estimates across studies and geographies.

## 10 Conclusions

Overall, research on the long-term health impacts and the economic burden of comorbid conditions associated with GD is needed to better understand the unmet treatment and other care needs of patients. Long-term registry studies and other real-world studies (e.g., chart reviews or database analyses) could be undertaken to address these evidence gaps. A cost-of-illness study, for example, could evaluate economic data from a variety of sources (e.g., insurance claims data, electronic health records, or patient surveys) to better understand the HCRU and costs associated with GD, including costs related to systemic manifestations associated with GD. Conducting a systematic review focused on specific aspects of the burden of GD would also provide a more robust evidence base. As novel targeted treatments are being investigated, data on the impacts of these treatments on comorbidities, HRQoL, and economic outcomes would be valuable. In summary, existing GD treatment options do not adequately address the full spectrum of GD manifestations, comorbidities, and long-term complications, highlighting the need for more effective therapies that improve long-term patient outcomes and HRQoL.
