# Supplementary material for: Graves’ disease: review of disease characteristics, burden, and treatments
Source: Front Endocrinol (Lausanne). 2026 Aug 18;17:1884624. doi: 10.3389/fendo.2026.1884624 (PMC13530974; doi:10.3389/fendo.2026.1884624)
Supplement: Supplementary file 1 [file Supplementaryfile1.docx]

Supplementary Material

# 1 Supplementary Tables

## 1.1 Supplementary Table 1. Treatments under investigation for TED/GO in ongoing, industry-sponsored Phase 3 trials registered on ClinicalTrials.gov as of June 12, 2025

Abbreviations: FcRn, neonatal fragment crystallizable receptor; GO, Graves’ ophthalmology; IGF-1R, insulin-like growth factor 1 receptor; IL-6, interleukin 6; IR, insulin receptor; mAb, monoclonal antibody; TED, thyroid eye disease

^a^ This study is sponsored by an academic institution, Peking University People's Hospital.

| Treatment | Sponsor | NCT Numbers | Locations | Type of Therapy |
| --- | --- | --- | --- | --- |
| Batoclimab | Immunovant, Inc. | - NCT05524571 - NCT05517421 - NCT05517447 | North America, Europe, and Oceania | Fully human anti-FcRn mAb |
| Efgartigimod | Argenx | - NCT06307626 - NCT06307613 | North America, Europe, and Asia | Antibody fragment targeting FcRn |
| IBI311 | Innovent Biologics (Suzhou) Co. Ltd. | - NCT06126783 - NCT06269393^a^ | China | Recombinant anti-IGF-1R antibody |
| MHB018A | Minghui Pharmaceutical (Hangzhou) Ltd | - [NCT06989918](https://clinicaltrials.gov/study/NCT06989918) | Not reported | Humanized anti-IGF1R antibody |
| Linsitinib | Sling Therapeutics, Inc. | - NCT05276063 - NCT06112340 | North America and Europe | Dual small-molecule kinase inhibitor of IGF-1R and IR |
| Satralizumab | Hoffmann-La Roche | - NCT05987423 - NCT06106828 | North America, South America, Europe, Asia, and Oceania | Recombinant, humanized anti-IL-6 receptor mAb |
| Teprotumumab | Amgen | - NCT06248619 | North America, Asia, and Oceania | Fully human anti-IGF-1R mAb |
| VRDN-001 (veligrotug) | Viridian Therapeutics, Inc. | - NCT06021054 - NCT06384547 - NCT06179875 - NCT05176639 | North America, Europe, and Oceania | Humanized anti-IGF-IR mAb |
| VRDN-003 | Viridian Therapeutics, Inc. | - NCT06625411 - NCT06625398 - NCT06812325 | United States | Humanized anti-IGF-IR mAb |

## 1.2 Supplementary Table 2. PICOS inclusion criteria

^a^ Conferences abstracts from the following conferences were hand searched: American Thyroid Association, British Thyroid Foundation, European Thyroid Association, International Society for Pharmacoeconomics and Outcomes Research.

Abbreviations: ATD, antithyroid drugs; ER, emergency room; HRQoL, health-related quality of life; ICU, intensive care unit; PRO, patient-reported outcome; QALY, quality-adjusted life year; UK, United Kingdom; US, United States.

| Criteria | Inclusion |
| --- | --- |
| Population | Adults (≥18 years) with Graves’ disease |
| Interventions/Comparators | Any approved or investigational treatment for Graves’ disease, including but not limited to ATD |
| Outcomes | Epidemiological Burden   - Incidence/prevalence of disease in the general population - Mortality - Risk factors - Proportion of patients experiencing specific symptoms, extrathyroidal manifestations, complications, and comorbidities - Diagnosis (criteria used for diagnosis and timing to diagnosis)   Clinical Burden (guidelines, treatments, treatment patterns, efficacy and safety)   - Clinical guideline recommendations - Treatments and treatment patterns - Summary of treatments in Phase 3 development - Outcome types recommended in guidelines and used in clinical trials - Brief summary of treatment efficacy and safety findings from systematic reviews, meta-analyses, network meta-analyses and key individual studies   Humanistic burden   - HRQoL/PRO scores - Utilities/disutilities   Economic Burden (costs and resource use)   - Total/direct/indirect costs of disease, treatment and complications - Resource use (hospitalizations, ICU stays, length of stays, ER visits) - Workdays lost/absenteeism, etc. - QALYs, life years gained, incremental costs associated with different treatments   Unmet Need   - Drivers of cost/resource use (healthcare, hospital, drug-related) - Unmet treatment needs |
| Study Design | Observational/real-world studies  Guidelines  SLRs/meta-analyses  Randomized controlled trials |
| Publication Type | Articles  Conference abstracts for selected conferences only^a^  Records from ClinicalTrials.gov |
| Date Limit | 2019 onwards for articles  2021 onwards for conference abstracts |
| Geography | Global, with the US, Canada, UK, and Europe as a priority |
| Language Limit | English |

## 1.3 Supplementary Table 3. Embase search strategy for clinical, epidemiological, and humanistic outcomes: Conducted via Ovid on August 2, 2024

*Trials filter based on*: Lefebvre C, Manheimer E, Glanville J. Chapter 6: searching for studies. 6.3.2.2. What is in The Cochrane Central Register of Controlled Trials (CENTRAL) from EMBASE? In: Higgins JPT, Green S (editors). Cochrane Handbook for Systematic Reviews of Interventions Version 5.1.0 [updated March 2011]. The Cochrane Collaboration, 2011. Available from https://handbook-5-1.cochrane.org/)

*Single arm search terms based on*: O'Rourke JM, Ward KE, Thompson JC. Single-Arm Trials in Systematic Reviews: Are They Adequately Captured by Existing Search Filters? *Value in Health*. 2016;19(7):A358.

*Systematic reviews filter*: SR / MA / HTA / ITC - MEDLINE, Embase, PsycInfo. In: CADTH Search Filters Database. Ottawa: CADTH; 2024: https://searchfilters.cadth.ca/link/33. Accessed 2024-08-02.

| Search Number | Search Terms | Results |
| --- | --- | --- |
| 1 | Graves disease/ | 25590 |
| 2 | ((graves$ or basedow$) adj3 (disease$ or syndrome$ or hyperthyroidism)).ti,ab. | 20993 |
| 3 | (exophthalmic adj3 (goiter or goitre)).ti,ab. | 21 |
| 4 | (autoimmune adj2 hyperthyroidism).ti,ab. | 513 |
| 5 | pretibial myxedema/ | 129 |
| 6 | ((graves$ or basedow$ or thyroid$ or infiltrative) adj3 (dermopath$ or acropach$)).ti,ab. | 209 |
| 7 | ((pretibial or tuberosum or local$) adj3 myx?edema).ti,ab. | 497 |
| 8 | endocrine ophthalmopathy/ | 7401 |
| 9 | ((graves$ or basedow$ or thyroid$ or dysthyroid$ or endocrine$ or congestive or edematous or oedematous or infiltrative or myopathic) adj3 ((eye$ adj3 disease$) or ophthalmic$ or ophthalmopath$ or orbitopath$)).ti,ab. | 8838 |
| 10 | or/1-9 | 35044 |
| 11 | crossover-procedure/ or double-blind procedure/ or randomized controlled trial/ or single-blind procedure/ | 923459 |
| 12 | (random$ or factorial$ or crossover$ or cross over$ or cross-over$ or placebo$ or (doubl$ adj blind$) or (singl$ adj blind$) or assign$ or allocat$ or volunteer$).ti,ab,ot. | 2976759 |
| 13 | single arm.ti,ab. | 32297 |
| 14 | (phase II trial$ or phase 2 trial$ or phase two trial$ or phase II study or phase 2 study or phase two study or phase II studies or phase 2 studies or phase two studies).ti,ab. | 80191 |
| 15 | (prospective trial$ or prospective study or prospective studies).ti,ab. | 365116 |
| 16 | or/11-15 | 3469508 |
| 17 | 10 and 16 | 2286 |
| 18 | *incidence/ | 42083 |
| 19 | *prevalence/ | 108550 |
| 20 | *mortality/ | 120915 |
| 21 | (incidence$ or prevalence$ or mortalit$).ti. or (incidence$ or prevalence$ or mortalit$).ab. /freq=3 | 1176932 |
| 22 | *risk factor/ | 138037 |
| 23 | (risk factor$ or relative risk$ or risk score$).ti. or (risk factor$ or relative risk$ or risk score$).ab. /freq=2 | 555636 |
| 24 | or/18-23 | 1674819 |
| 25 | 10 and 24 | 1593 |
| 26 | treatment duration/ | 310529 |
| 27 | drug dose/ | 31080 |
| 28 | ((treatment$ or therapeutic or therapy or therapies) adj2 (pattern$ or pathway$ or trend$ or utili$ or management or prevalence or initiat$ or duration or distribution$ or discontin$ or coverage or variety or selection or spread)).ti,ab. | 396461 |
| 29 | ((medication$ or medicating or drug or drugs or prescrip$ or prescrib$) adj2 (pattern$ or pathways$ or trend$ or utili$ or management or prevalence or initiat$ or duration or distribution$ or discontin$ or coverage or variety or selection or spread)).ti,ab. | 127422 |
| 30 | unmet medical need/ or ((unmet or "not met" or unfulfilled or "not fulfilled" or unachieved or "not achieved") adj3 (need or needs or demand$)).ti,ab. | 63930 |
| 31 | or/26-30 | 849741 |
| 32 | 10 and 31 | 1693 |
| 33 | practice guideline/ or clinical pathway/ or clinical protocol/ or consensus development/ or nursing protocol/ | 731431 |
| 34 | guideline$.ti. | 133475 |
| 35 | guidance.ti,ab. | 250544 |
| 36 | care pathway$.ti,ab. | 14067 |
| 37 | (clinical adj3 pathway$).ti,ab. | 15283 |
| 38 | (practice adj3 parameter$).ti,ab. | 2744 |
| 39 | meta-analysis/ or systematic review/ or "meta analysis (topic)"/ or "systematic review (topic)"/ or exp biomedical technology assessment/ or network meta-analysis/ | 699559 |
| 40 | ((systematic* adj3 (review* or overview*)) or (methodologic* adj3 (review* or overview*))).ti,ab,kf. | 454375 |
| 41 | ((quantitative adj3 (review* or overview* or synthes*)) or (research adj3 (integrati* or overview*))).ti,ab,kf. | 20602 |
| 42 | ((integrative adj3 (review* or overview*)) or (collaborative adj3 (review* or overview*)) or (pool* adj3 analy*)).ti,ab,kf. | 61002 |
| 43 | (data synthes* or data extraction* or data abstraction*).ti,ab,kf. | 56575 |
| 44 | (handsearch* or hand search*).ti,ab,kf. | 14265 |
| 45 | (mantel haenszel or peto or der simonian or dersimonian or fixed effect* or latin square*).ti,ab,kf. | 51706 |
| 46 | (met analy* or metanaly* or technology assessment* or HTA or HTAs or technology overview* or technology appraisal*).ti,ab,kf. | 22562 |
| 47 | (meta regression* or metaregression*).ti,ab,kf. | 20602 |
| 48 | (meta-analy* or metaanaly* or systematic review* or biomedical technology assessment* or bio-medical technology assessment*).mp,hw. | 827949 |
| 49 | (medline or cochrane or pubmed or medlars or embase or cinahl).ti,ab,hw. | 508664 |
| 50 | (cochrane or (health adj2 technology assessment) or evidence report).jw. | 32293 |
| 51 | (comparative adj3 (efficacy or effectiveness)).ti,ab,kf. | 28512 |
| 52 | (outcomes research or relative effectiveness).ti,ab,kf. | 17127 |
| 53 | ((indirect or indirect treatment or mixed-treatment or bayesian) adj3 comparison*).ti,ab,kf. | 8249 |
| 54 | (multi* adj3 treatment adj3 comparison*).ti,ab,kf. | 450 |
| 55 | (mixed adj3 treatment adj3 (meta-analy* or metaanaly*)).ti,ab,kf. | 263 |
| 56 | umbrella review*.ti,ab,kf. | 2273 |
| 57 | (multi* adj2 paramet* adj2 evidence adj2 synthesis).ti,ab,kf. | 35 |
| 58 | (multiparamet* adj2 evidence adj2 synthesis).ti,ab,kf. | 22 |
| 59 | (multi-paramet* adj2 evidence adj2 synthesis).ti,ab,kf. | 30 |
| 60 | or/33-59 | 2024237 |
| 61 | 10 and 60 | 1742 |
| 62 | productivity/ or absenteeism/ or caregiver burden/ or caregiver/ or work disability/ or "quality of life"/ | 856379 |
| 63 | patient satisfaction/ or satisfaction/ or life satisfaction/ or patient preference/ or daily life activity/ | 412966 |
| 64 | patient-reported outcome/ | 64154 |
| 65 | (quality of life or life quality or qol or hrqol or activities of daily living).ti,ab. | 702632 |
| 66 | (patient satisfaction or life satisfaction).ti,ab. | 85184 |
| 67 | (patient reported outcome$ or patient reported treatment or self reported outcome$ or PROs or PROMs).ti,ab. | 83094 |
| 68 | ((human$ or social$ or societ$ or work$ or employe$ or business$ or communit$ or famil$ or carer$ or caregiver$) adj3 (burden$ or consequenc$ or impact$ or problem$ or productivity or sickness or impairment$)).ti,ab. | 224760 |
| 69 | ((long standing or longstanding or long term or longterm or permanent or employee$) adj2 (absence$ or absent$ or ill$ or sick$ or disab$)).ti,ab. | 19734 |
| 70 | (llsi or ((emergenc$ or domestic$ or famil$ or carer$ or caregiver$) adj3 leave$)).ti,ab. | 1561 |
| 71 | (burden adj2 (illness$ or disease$ or sickness$)).ti,ab. | 66486 |
| 72 | ((allowance or status or long-term or pension$ or benefit$) adj2 (disab$ or incapacit$)).ti,ab. | 29062 |
| 73 | ((unable or inability or incapacit$ or incapab$) adj3 work).ti,ab. | 3460 |
| 74 | ((resource$ or fund$) adj2 (use$ or utili?ation)).ti,ab. | 72259 |
| 75 | ((health or healthcare) adj2 (resource$ or fund$)).ti,ab. | 66287 |
| 76 | ("length of stay" or "duration of stay" or "extended stay" or "prolonged stay").ti,ab. | 166762 |
| 77 | ((ambulatory or ambulance or hospital or A&E or emergency) adj2 (attention$ or trip or trips or visit$ or stay$ or admission$ or admitted or transport$)).ti,ab. | 382140 |
| 78 | ((GP or general practitioner$ or doctor$ or clinician$ or specialist$ or physician$) adj2 (appointment$ or attention$ or trip or trips or visit$)).ti,ab. | 26229 |
| 79 | (in-patient stay$ or inpatient stay$).ti,ab. | 8206 |
| 80 | or/62-79 | 2248853 |
| 81 | 10 and 80 | 1641 |
| 82 | animal/ or animal experiment/ or (rat or rats or mouse or mice or murine or rodent or rodents or hamster or hamsters or pig or pigs or porcine or rabbit or rabbits or animal or animals or dogs or dog or cats or cow or bovine or sheep or ovine or monkey or monkeys).ti,ab,ot,hw. | 7913835 |
| 83 | exp human/ or human experiment/ | 26932405 |
| 84 | 82 not (82 and 83) | 5890203 |
| 85 | case reports/ or case study/ or case report$.jx. or case report$.jw. or (case report or case study or case series).ti. | 848654 |
| 86 | (news or "newspaper article" or editorial or comment or letter or overall or short survey or tombstone or books).pt. or in vitro Techniques/ or in vitro study/ or (commentary or editorial or comment or letter or mice or rat or mouse or animal or murine).ti. | 5498577 |
| 87 | ("conference abstract" or "conference review" or "conference paper").pt. or conference$.so,st. | 6029156 |
| 88 | or/84-87 | 15500102 |
| 89 | 17 not 88 | 1756 |
| 90 | 25 not 88 | 1160 |
| 91 | 32 not 88 | 1089 |
| 92 | 61 not 88 | 1271 |
| 93 | 81 not 88 | 1082 |
| 94 | limit 89 to yr="2019 -Current" [Clinical and safety] | 566 |
| 95 | limit 90 to yr="2019 -Current" [Epidemiology and Risk Factors] | 398 |
| 96 | limit 91 to yr="2019 -Current" [Treatment patterns and unmet needs] | 341 |
| 97 | limit 92 to yr="2019 -Current" [Guidelines and systematic reviews] | 611 |
| 98 | limit 93 to yr="2019 -Current" [Humanistic burden] | 481 |
| 99 | 94 or 95 or 96 or 97 or 98 [All Topics Combined] | 1856 |

## 1.4 Supplementary Table 4. MEDLINE search strategy for clinical, epidemiological, and humanistic outcomes: Conducted via Ovid on August 2, 2024

*Trials filter based on*: Box 3.d Cochrane Highly Sensitive Search Strategy for identifying randomized trials in MEDLINE: sensitivity- and precision-maximizing version (2008 revision); Ovid format. Lefebvre C, Glanville J, Briscoe S, Featherstone R, Littlewood A, Marshall C, Metzendorf M-I, Noel-Storr A, Paynter R, Rader T, Thomas J, Wieland LS. Technical Supplement to Chapter 4: Searching for and selecting studies. In: Higgins JPT, Thomas J, Chandler J, Cumpston MS, Li T, Page MJ, Welch VA (eds). Cochrane Handbook for Systematic Reviews of Interventions Version 6.3 (updated February 2022). Cochrane, 2022. Available from: www.training.cochrane.org/handbook.

*Single arm search terms based on*: O'Rourke JM, Ward KE, Thompson JC. Single-Arm Trials in Systematic Reviews: Are They Adequately Captured by Existing Search Filters? *Value in Health*. 2016;19(7):A358

*Systematic reviews filter*: SR / MA / HTA / ITC - MEDLINE, Embase, PsycInfo. In: CADTH Search Filters Database. Ottawa: CADTH; 2024: https://searchfilters.cadth.ca/link/33. Accessed 2024-08-02.

| Search Number | Search Terms | Results |
| --- | --- | --- |
| 1 | exp Graves Disease/ | 19322 |
| 2 | ((graves$ or basedow$) adj3 (disease$ or syndrome$ or hyperthyroidism)).ti,ab. | 16054 |
| 3 | (exophthalmic adj3 (goiter or goitre)).ti,ab. | 272 |
| 4 | (autoimmune adj2 hyperthyroidism).ti,ab. | 356 |
| 5 | Myxedema/ | 3832 |
| 6 | ((graves$ or basedow$ or thyroid$ or infiltrative) adj3 (dermopath$ or acropach$)).ti,ab. | 168 |
| 7 | ((pretibial or tuberosum or local$) adj3 myx?edema).ti,ab. | 498 |
| 8 | Graves Ophthalmopathy/ | 3338 |
| 9 | ((graves$ or basedow$ or thyroid$ or dysthyroid$ or endocrine$ or congestive or edematous or oedematous or infiltrative or myopathic) adj3 ((eye$ adj3 disease$) or ophthalmic$ or ophthalmopath$ or orbitopath$)).ti,ab. | 6893 |
| 10 | or/1-9 | 29353 |
| 11 | randomized controlled trial.pt. | 618186 |
| 12 | controlled clinical trial.pt. | 95583 |
| 13 | randomized.ab. | 655132 |
| 14 | placebo.ab. | 250533 |
| 15 | clinical trials as topic.sh. | 202925 |
| 16 | randomly.ab. | 439045 |
| 17 | trial.ti. | 314764 |
| 18 | single arm.ti,ab. | 15450 |
| 19 | (phase II trial$ or phase 2 trial$ or phase two trial$ or phase II study or phase 2 study or phase two study or phase II studies or phase 2 studies or phase two studies).ti,ab. | 39220 |
| 20 | (prospective trial$ or prospective study or prospective studies).ti,ab. | 234329 |
| 21 | or/11-20 | 1845926 |
| 22 | 10 and 21 | 1180 |
| 23 | *Incidence/ | 610 |
| 24 | *Prevalence/ | 947 |
| 25 | exp *Mortality/ | 72764 |
| 26 | (incidence$ or prevalence$ or mortalit$).ti. or (incidence$ or prevalence$ or mortalit$).ab. /freq=2 | 1251939 |
| 27 | *Risk Factors/ | 1293 |
| 28 | (risk factor$ or relative risk$ or risk score$).ti. or (risk factor$ or relative risk$ or risk score$).ab. /freq=2 | 379732 |
| 29 | or/23-28 | 1548643 |
| 30 | 10 and 29 | 1328 |
| 31 | "Duration of Therapy"/ | 753 |
| 32 | ((treatment$ or therapeutic or therapy or therapies) adj2 (pattern$ or pathway$ or trend$ or utili$ or management or prevalence or initiat$ or duration or distribution$ or discontin$ or coverage or variety or selection or spread)).ti,ab. | 232010 |
| 33 | ((medication$ or medicating or drug or drugs or prescrip$ or prescrib$) adj2 (pattern$ or pathways$ or trend$ or utili$ or management or prevalence or initiat$ or duration or distribution$ or discontin$ or coverage or variety or selection or spread)).ti,ab. | 78416 |
| 34 | "Health Services Needs and Demand"/ or ((unmet or "not met" or unfulfilled or "not fulfilled" or unachieved or "not achieved") adj3 (need or needs or demand$)).ti,ab. | 90648 |
| 35 | or/31-34 | 391664 |
| 36 | 10 and 35 | 610 |
| 37 | Guidelines as Topic/ or Practice Guidelines as Topic/ or Health Planning Guidelines/ or Practice Guideline/ | 205713 |
| 38 | guideline$.ti. | 99055 |
| 39 | guidance.ti,ab. | 176649 |
| 40 | care pathway$.ti,ab. | 8226 |
| 41 | (clinical adj3 pathway$).ti,ab. | 9360 |
| 42 | (practice adj3 parameter$).ti,ab. | 1877 |
| 43 | (systematic review or meta-analysis).pt. | 356540 |
| 44 | Systematic Review/ or Meta-Analysis/ or Systematic Reviews as Topic/ or Meta-Analysis as Topic/ or exp Technology Assessment, Biomedical/ or Network Meta-Analysis/ | 398746 |
| 45 | ((systematic* adj3 (review* or overview*)) or (methodologic* adj3 (review* or overview*))).ti,ab,kf. | 374279 |
| 46 | ((quantitative adj3 (review* or overview* or synthes*)) or (research adj3 (integrati* or overview*))).ti,ab,kf. | 17847 |
| 47 | ((integrative adj3 (review* or overview*)) or (collaborative adj3 (review* or overview*)) or (pool* adj3 analy*)).ti,ab,kf. | 43347 |
| 48 | (data synthes* or data extraction* or data abstraction*).ti,ab,kf. | 46337 |
| 49 | (handsearch* or hand search*).ti,ab,kf. | 11689 |
| 50 | (mantel haenszel or peto or der simonian or dersimonian or fixed effect* or latin square*).ti,ab,kf. | 39050 |
| 51 | (met analy* or metanaly* or technology assessment* or HTA or HTAs or technology overview* or technology appraisal*).ti,ab,kf. | 13262 |
| 52 | (meta regression* or metaregression*).ti,ab,kf. | 16818 |
| 53 | (meta-analy* or metaanaly* or systematic review* or biomedical technology assessment* or bio-medical technology assessment*).mp,hw. | 531216 |
| 54 | (medline or cochrane or pubmed or medlars or embase or cinahl).ti,ab,hw. | 390890 |
| 55 | (cochrane or (health adj2 technology assessment) or evidence report).jw. | 21993 |
| 56 | (comparative adj3 (efficacy or effectiveness)).ti,ab,kf. | 19386 |
| 57 | (outcomes research or relative effectiveness).ti,ab,kf. | 11845 |
| 58 | ((indirect or indirect treatment or mixed-treatment or bayesian) adj3 comparison*).ti,ab,kf. | 4702 |
| 59 | (multi* adj3 treatment adj3 comparison*).ti,ab,kf. | 312 |
| 60 | (mixed adj3 treatment adj3 (meta-analy* or metaanaly*)).ti,ab,kf. | 181 |
| 61 | umbrella review*.ti,ab,kf. | 2143 |
| 62 | (multi* adj2 paramet* adj2 evidence adj2 synthesis).ti,ab,kf. | 14 |
| 63 | (multiparamet* adj2 evidence adj2 synthesis).ti,ab,kf. | 19 |
| 64 | (multi-paramet* adj2 evidence adj2 synthesis).ti,ab,kf. | 12 |
| 65 | or/37-64 | 1167308 |
| 66 | 10 and 65 | 767 |
| 67 | absenteeism/ | 9903 |
| 68 | caregivers/ | 53914 |
| 69 | "Quality of Life"/ | 291452 |
| 70 | Personal Satisfaction/ or exp Patient Satisfaction/ or "Activities of Daily Living"/ | 198154 |
| 71 | Patient Reported Outcome Measures/ | 15730 |
| 72 | (quality of life or life quality or qol or hrqol or activities of daily living).ti,ab. | 447996 |
| 73 | (patient satisfaction or life satisfaction).ti,ab. | 61032 |
| 74 | (patient reported outcome$ or patient reported treatment or self reported outcome$ or PROs or PROMs).ti,ab. | 51992 |
| 75 | ((human$ or social$ or societ$ or work$ or employe$ or business$ or communit$ or famil$ or carer$ or caregiver$) adj3 (burden$ or consequenc$ or impact$ or problem$ or productivity or sickness or impairment$)).ti,ab. | 173977 |
| 76 | ((long standing or longstanding or long term or longterm or permanent or employee$) adj2 (absence$ or absent$ or ill$ or sick$ or disab$)).ti,ab. | 14123 |
| 77 | (llsi or ((emergenc$ or domestic$ or famil$ or carer$ or caregiver$) adj3 leave$)).ti,ab. | 1259 |
| 78 | (burden adj2 (illness$ or disease$ or sickness$)).ti,ab. | 43178 |
| 79 | ((allowance or status or long-term or pension$ or benefit$) adj2 (disab$ or incapacit$)).ti,ab. | 17934 |
| 80 | ((unable or inability or incapacit$ or incapab$) adj3 work).ti,ab. | 2304 |
| 81 | ((resource$ or fund$) adj2 (use$ or utili?ation)).ti,ab. | 46812 |
| 82 | ((health or healthcare) adj2 (resource$ or fund$)).ti,ab. | 44285 |
| 83 | ("length of stay" or "duration of stay" or "extended stay" or "prolonged stay").ti,ab. | 88756 |
| 84 | ((ambulatory or ambulance or hospital or A&E or emergency) adj2 (attention$ or trip or trips or visit$ or stay$ or admission$ or admitted or transport$)).ti,ab. | 231026 |
| 85 | ((GP or general practitioner$ or doctor$ or clinician$ or specialist$ or physician$) adj2 (appointment$ or attention$ or trip or trips or visit$)).ti,ab. | 16595 |
| 86 | (in-patient stay$ or inpatient stay$).ti,ab. | 4011 |
| 87 | or/67-86 | 1327338 |
| 88 | 10 and 87 | 727 |
| 89 | exp animals/ not humans/ | 5244920 |
| 90 | case reports/ or case study/ or case report$.jw. or (case report or case series or woman or man or child or adolescent or female or male or boy or girl or infant).ti. | 3019881 |
| 91 | ("Introductory Journal Article" or letter or News or "Newspaper Article" or Editorial or Comment or Overall).pt. or in vitro Techniques/ or (commentary or editorial or comment or letter).ti. | 3012422 |
| 92 | or/89-91 | 10561031 |
| 93 | 22 not 92 | 1125 |
| 94 | 30 not 92 | 1233 |
| 95 | 36 not 92 | 434 |
| 96 | 66 not 92 | 690 |
| 97 | 88 not 92 | 636 |
| 98 | limit 93 to yr="2019 -Current" [Clinical and safety] | 271 |
| 99 | limit 94 to yr="2019 -Current" [Epidemiology and Risk Factors] | 313 |
| 100 | limit 95 to yr="2019 -Current" [Treatment patterns and unmet needs] | 115 |
| 101 | limit 96 to yr="2019 -Current" [Guidelines and systematic reviews] | 319 |
| 102 | limit 97 to yr="2019 -Current" [Humanistic burden] | 269 |
| 103 | 98 or 99 or 100 or 101 or 102 [All Topics Combined] | 1044 |

## 1.5 Supplementary Table 5. CENTRAL (Cochrane Central Register of Controlled Trials. EBM Reviews) search strategy for clinical, epidemiological, and humanistic outcomes: Conducted via Ovid on August 2, 2024

| Search Number | Search Terms | Results |
| --- | --- | --- |
| 1 | exp Graves Disease/ | 588 |
| 2 | ((graves$ or basedow$) adj3 (disease$ or syndrome$ or hyperthyroidism)).ti,ab. | 643 |
| 3 | (exophthalmic adj3 (goiter or goitre)).ti,ab. | 2 |
| 4 | (autoimmune adj2 hyperthyroidism).ti,ab. | 11 |
| 5 | Myxedema/ | 5 |
| 6 | ((graves$ or basedow$ or thyroid$ or infiltrative) adj3 (dermopath$ or acropach$)).ti,ab. | 3 |
| 7 | ((pretibial or tuberosum or local$) adj3 myx?edema).ti,ab. | 5 |
| 8 | Graves Ophthalmopathy/ | 192 |
| 9 | ((graves$ or basedow$ or thyroid$ or dysthyroid$ or endocrine$ or congestive or edematous or oedematous or infiltrative or myopathic) adj3 ((eye$ adj3 disease$) or ophthalmic$ or ophthalmopath$ or orbitopath$)).ti,ab. | 534 |
| 10 | or/1-9 | 1083 |
| 11 | trial registry record.pt. | 528251 |
| 12 | conference proceeding.pt. | 242948 |
| 13 | 11 or 12 | 771199 |
| 14 | 10 not 13 | 680 |
| 15 | limit 14 to yr="2019 -Current" | 122 |

## 1.6 Supplementary Table 6. CDSR (Cochrane Database of Systematic Reviews. EBM Reviews) search strategy for clinical, epidemiological, and humanistic outcomes: Conducted via Ovid on August 2, 2024

| Search Number | Search Terms | Results |
| --- | --- | --- |
| 1 | ((graves$ or basedow$) adj3 (disease$ or syndrome$ or hyperthyroidism)).ti,ab,kw. | 12 |
| 2 | (exophthalmic adj3 (goiter or goitre)).ti,ab,kw. | 0 |
| 3 | (autoimmune adj2 hyperthyroidism).ti,ab,kw. | 0 |
| 4 | ((graves$ or basedow$ or thyroid$ or infiltrative) adj3 (dermopath$ or acropach$)).ti,ab,kw. | 0 |
| 5 | ((pretibial or tuberosum or local$) adj3 myx?edema).ti,ab,kw. | 0 |
| 6 | ((graves$ or basedow$ or thyroid$ or dysthyroid$ or endocrine$ or congestive or edematous or oedematous or infiltrative or myopathic) adj3 ((eye$ adj3 disease$) or ophthalmic$ or ophthalmopath$ or orbitopath$)).ti,ab,kw. | 7 |
| 7 | or/1-6 | 13 |

## 1.7 Supplementary Table 7. Embase search strategy for economic outcomes: Conducted via OVID on August 2, 2024

*Utilities filter translated from*: Health state utility values/HRQoL. Arber M, Garcia S, Veale T, Edwards M, Shaw A, Glanville JM. Performance of Ovid Medline search filters to identify health state utility studies. *Int J Technol Assess Health Care* 2017;33(4):472-80

| Search Number | Search Terms | Results |
| --- | --- | --- |
| 1 | Graves disease/ | 25590 |
| 2 | ((graves$ or basedow$) adj3 (disease$ or syndrome$ or hyperthyroidism)).ti,ab. | 20993 |
| 3 | (exophthalmic adj3 (goiter or goitre)).ti,ab. | 21 |
| 4 | (autoimmune adj2 hyperthyroidism).ti,ab. | 513 |
| 5 | pretibial myxedema/ | 129 |
| 6 | ((graves$ or basedow$ or thyroid$ or infiltrative) adj3 (dermopath$ or acropach$)).ti,ab. | 209 |
| 7 | ((pretibial or tuberosum or local$) adj3 myx?edema).ti,ab. | 497 |
| 8 | endocrine ophthalmopathy/ | 7401 |
| 9 | ((graves$ or basedow$ or thyroid$ or dysthyroid$ or endocrine$ or congestive or edematous or oedematous or infiltrative or myopathic) adj3 ((eye$ adj3 disease$) or ophthalmic$ or ophthalmopath$ or orbitopath$)).ti,ab. | 8838 |
| 10 | or/1-9 | 35044 |
| 11 | economic evaluation/ or cost utility analysis/ or cost benefit analysis/ or cost minimization analysis/ or cost effectiveness analysis/ or quality adjusted life year/ | 303655 |
| 12 | (cost$ adj3 (utilit$ or effective$ or assess$ or evaluat$ or analys$ or model$ or benefit$ or minimi$ or threshold$ or outcome or outcomes)).ti,ab. | 367241 |
| 13 | (economic$ adj3 (evaluat$ or assess$ or analys$ or model$ or benefit$ or minimi$ or threshold$ or outcome or outcomes)).ti,ab. | 75874 |
| 14 | (CEA or CUA or CBA).ti,ab. | 59707 |
| 15 | exp Monte Carlo method/ | 56277 |
| 16 | Markov chain/ | 11829 |
| 17 | "decision tree"/ | 25402 |
| 18 | (markov adj3 (chain$ or model$ or economic$)).ti,ab. | 34192 |
| 19 | monte carlo.ti,ab. | 64987 |
| 20 | (decision adj2 (tree$ or analy$ or model$)).ti,ab. | 55340 |
| 21 | statistical model/ | 178568 |
| 22 | statistical model$.ti,ab. | 26022 |
| 23 | ((incremental adj2 cost$) or icer).ti,ab. | 32846 |
| 24 | (quality adjusted life$ or QALY$).ti,ab. | 35401 |
| 25 | or/11-24 | 914079 |
| 26 | exp health care cost/ | 356522 |
| 27 | health care utilization/ | 102163 |
| 28 | "cost"/ | 64837 |
| 29 | (econom$ or cost or costs or costly or costing or price or prices or pricing or pharmacoeconomic$).ti,ab. | 1495660 |
| 30 | ((healthcare or health care or health resource or medical resource) adj3 ("use" or utili?ation)).ti,ab. | 76670 |
| 31 | (HCRU or MRU).ti,ab. | 3016 |
| 32 | ((economic or health$ or resource) adj3 burden).ti,ab. | 66577 |
| 33 | (expenditure$ not energy).ti,ab. | 53506 |
| 34 | (value adj2 money).ti,ab. | 3152 |
| 35 | budget$.ti,ab. | 49981 |
| 36 | or/26-35 | 1825083 |
| 37 | ((metabolic adj cost) or (energy adj cost) or (energy adj expenditure)).ti,ab. | 44286 |
| 38 | 36 not 37 | 1816613 |
| 39 | quality adjusted life year/ | 38137 |
| 40 | (quality adjusted or adjusted life year$).ti,ab. | 37217 |
| 41 | (qaly$ or qald$ or qale$ or qtime$).ti,ab. | 28871 |
| 42 | (illness state$1 or health state$1).ti,ab. | 15582 |
| 43 | (hui or hui1 or hui2 or hui3).ti,ab. | 3379 |
| 44 | (multiattribute$ or multi attribute$).ti,ab. | 1621 |
| 45 | (utility adj3 (score$1 or valu$ or health$ or cost$ or measur$ or disease$ or mean or gain or gains or index$)).ti,ab. | 33724 |
| 46 | utilities.ti,ab. | 15967 |
| 47 | (eq-5d or eq5d or eq-5 or eq5 or euro qual or euroqual or euro qual5d or euroqual5d or euro qol or euroqol or euro qol5d or euroqol5d or euro quol or euroquol or euro quol5d or euroquol5d or eur qol or eurqol or eur qol5d or eur qol5d or eur?qul or eur?qul5d or euro$ quality of life or european qol).ti,ab. | 34126 |
| 48 | (euro$ adj3 (5 d or 5d or 5 dimension$ or 5dimension$ or 5 domain$ or 5domain$)).ti,ab. | 9782 |
| 49 | (sf36$ or sf 36$ or sf thirtysix or sf thirty six).ti,ab. | 47554 |
| 50 | (time trade off$1 or time tradeoff$1 or tto or timetradeoff$1).ti,ab. | 3727 |
| 51 | or/39-50 | 164586 |
| 52 | 10 and (25 or 38 or 51) | 833 |
| 53 | animal/ or animal experiment/ or (rat or rats or mouse or mice or murine or rodent or rodents or hamster or hamsters or pig or pigs or porcine or rabbit or rabbits or animal or animals or dogs or dog or cats or cow or bovine or sheep or ovine or monkey or monkeys).ti,ab,ot,hw. | 7913835 |
| 54 | exp human/ or human experiment/ | 26932405 |
| 55 | 53 not (53 and 54) | 5890203 |
| 56 | case reports/ or case study/ or case report$.jx. or case report$.jw. or (case report or case study or case series).ti. | 848654 |
| 57 | (news or "newspaper article" or editorial or comment or letter or overall or short survey or tombstone or books).pt. or in vitro Techniques/ or in vitro study/ or (commentary or editorial or comment or letter or mice or rat or mouse or animal or murine).ti. | 5498577 |
| 58 | ("conference abstract" or "conference review" or "conference paper").pt. or conference$.so,st. | 6029156 |
| 59 | or/55-58 | 15500102 |
| 60 | 52 not 59 | 609 |
| 61 | limit 60 to yr="2019 -Current" [Economic] | 195 |

## 1.8 Supplementary Table 8. MEDLINE search strategy for economic outcomes: Conducted via OVID on August 2, 2024

*Utilities filter*: Health state utility values/HRQoL. Arber M, Garcia S, Veale T, Edwards M, Shaw A, Glanville JM. Performance of Ovid Medline search filters to identify health state utility studies. *Int J Technol Assess Health Care* 2017;33(4):472-80

| Search Number | Search Terms | Results |
| --- | --- | --- |
| 1 | exp Graves Disease/ | 19322 |
| 2 | ((graves$ or basedow$) adj3 (disease$ or syndrome$ or hyperthyroidism)).ti,ab. | 16054 |
| 3 | (exophthalmic adj3 (goiter or goitre)).ti,ab. | 272 |
| 4 | (autoimmune adj2 hyperthyroidism).ti,ab. | 356 |
| 5 | Myxedema/ | 3832 |
| 6 | ((graves$ or basedow$ or thyroid$ or infiltrative) adj3 (dermopath$ or acropach$)).ti,ab. | 168 |
| 7 | ((pretibial or tuberosum or local$) adj3 myx?edema).ti,ab. | 498 |
| 8 | Graves Ophthalmopathy/ | 3338 |
| 9 | ((graves$ or basedow$ or thyroid$ or dysthyroid$ or endocrine$ or congestive or edematous or oedematous or infiltrative or myopathic) adj3 ((eye$ adj3 disease$) or ophthalmic$ or ophthalmopath$ or orbitopath$)).ti,ab. | 6893 |
| 10 | or/1-9 | 29353 |
| 11 | Cost-Benefit Analysis/ | 95283 |
| 12 | disability-adjusted life years/ or quality-adjusted life years/ | 16878 |
| 13 | (cost$ adj3 (utilit$ or effective$ or assess$ or evaluat$ or analys$ or model$ or benefit$ or minimi$ or threshold$ or outcome or outcomes)).ti,ab. | 267808 |
| 14 | (economic$ adj3 (evaluat$ or assess$ or analys$ or model$ or benefit$ or minimi$ or threshold$ or outcome or outcomes)).ti,ab. | 54884 |
| 15 | (CEA or CUA or CBA).ti,ab. | 40854 |
| 16 | monte carlo method/ | 33117 |
| 17 | markov chains/ | 16331 |
| 18 | Decision Trees/ | 12293 |
| 19 | (markov adj3 (chain$ or model$ or economic$)).ti,ab. | 25130 |
| 20 | monte carlo.ti,ab. | 62163 |
| 21 | (decision adj2 (tree$ or analy$ or model$)).ti,ab. | 41508 |
| 22 | exp models, statistical/ | 459869 |
| 23 | statistical model$.ti,ab. | 21835 |
| 24 | ((incremental adj2 cost$) or icer).ti,ab. | 19278 |
| 25 | (quality adjusted life$ or QALY$).ti,ab. | 20985 |
| 26 | or/11-25 | 940388 |
| 27 | 10 and 26 | 345 |
| 28 | exp Health Care Costs/ | 73418 |
| 29 | "costs and cost analysis"/ or exp "cost control"/ or exp "cost of illness"/ or exp health expenditures/ | 140035 |
| 30 | (econom$ or cost or costs or costly or costing or price or prices or pricing or pharmacoeconomic$).ti,ab. | 1172929 |
| 31 | ((healthcare or health care or health resource or medical resource) adj3 ("use" or utili?ation)).ti,ab. | 49628 |
| 32 | (HCRU or MRU).ti,ab. | 1191 |
| 33 | ((economic or health$ or resource) adj3 burden).ti,ab. | 44287 |
| 34 | (expenditure$ not energy).ti,ab. | 39337 |
| 35 | (value adj2 money).ti,ab. | 2300 |
| 36 | budget$.ti,ab. | 37881 |
| 37 | or/28-36 | 1333376 |
| 38 | ((metabolic adj cost) or (energy adj cost) or (energy adj expenditure)).ti,ab. | 35375 |
| 39 | 37 not 38 | 1325525 |
| 40 | 10 and 39 | 328 |
| 41 | quality-adjusted life years/ | 16638 |
| 42 | (quality adjusted or adjusted life year$).ti,ab. | 25583 |
| 43 | (qaly$ or qald$ or qale$ or qtime$).ti,ab. | 15615 |
| 44 | (illness state$1 or health state$1).ti,ab. | 8885 |
| 45 | (hui or hui1 or hui2 or hui3).ti,ab. | 2107 |
| 46 | (multiattribute$ or multi attribute$).ti,ab. | 1426 |
| 47 | (utility adj3 (score$1 or valu$ or health$ or cost$ or measur$ or disease$ or mean or gain or gains or index$)).ti,ab. | 21374 |
| 48 | utilities.ti,ab. | 9967 |
| 49 | (eq-5d or eq5d or eq-5 or eq5 or euro qual or euroqual or euro qual5d or euroqual5d or euro qol or euroqol or euro qol5d or euroqol5d or euro quol or euroquol or euro quol5d or euroquol5d or eur qol or eurqol or eur qol5d or eur qol5d or eur?qul or eur?qul5d or euro$ quality of life or european qol).ti,ab. | 19197 |
| 50 | (euro$ adj3 (5 d or 5d or 5 dimension$ or 5dimension$ or 5 domain$ or 5domain$)).ti,ab. | 6545 |
| 51 | (sf36$ or sf 36$ or sf thirtysix or sf thirty six).ti,ab. | 27769 |
| 52 | (time trade off$1 or time tradeoff$1 or tto or timetradeoff$1).ti,ab. | 2497 |
| 53 | or/41-52 | 100840 |
| 54 | 10 and 53 | 47 |
| 55 | 27 or 40 or 54 | 579 |
| 56 | exp animals/ not humans/ | 5244920 |
| 57 | case reports/ or case study/ or case report$.jw. or (case report or case series).ti. | 2518175 |
| 58 | ("Introductory Journal Article" or letter or News or "Newspaper Article" or Editorial or Comment or Overall).pt. or in vitro Techniques/ or (commentary or editorial or comment or letter or mice or rat or mouse or animal or murine).ti. | 4180677 |
| 59 | or/56-58 | 10422412 |
| 60 | 55 not 59 | 537 |
| 61 | limit 60 to yr="2019 -Current" [Economic] | 138 |

## 1.9 Supplementary Table 9. Econlit Search Strategy for Economic Outcomes: Conducted via OVID on August 2, 2024

| Search Number | Search Terms | Results |
| --- | --- | --- |
| 1 | ((graves$ or basedow$) adj3 (disease$ or syndrome$ or hyperthyroidism)).ti,ab. | 0 |
| 2 | (exophthalmic adj3 (goiter or goitre)).ti,ab. | 0 |
| 3 | (autoimmune adj2 hyperthyroidism).ti,ab. | 0 |
| 4 | ((graves$ or basedow$ or thyroid$ or infiltrative) adj3 (dermopath$ or acropach$)).ti,ab. | 0 |
| 5 | ((pretibial or tuberosum or local$) adj3 myx?edema).ti,ab. | 0 |
| 6 | ((graves$ or basedow$ or thyroid$ or dysthyroid$ or endocrine$ or congestive or edematous or oedematous or infiltrative or myopathic) adj3 ((eye$ adj3 disease$) or ophthalmic$ or ophthalmopath$ or orbitopath$)).ti,ab. | 0 |
| 7 | or/1-6 | 0 |

# 2 Supplementary Figures

## 2.1 Supplementary Figure 1. Targeted literature review flow diagram

^a^ Records that were from non-target countries (e.g., Iran, China, Thailand) were prioritized for inclusion if they addressed evidence gaps.

Abbreviations: PICOS, population, interventions and comparisons, outcomes, and study design; UK, United Kingdom; US, United States.
